# The Physiological Practitioner

**Published:** 1846-11

**Authors:** 


*The Physiological Practitioner.*—The following extract occurs in a communication to the Editor of the British and Foreign Medical Review. We find it in the New Orleans Med. and Surg. Journal. We quote it not alone for its piquancy of style, but for the important truth which it contains. We have in this country, also, writers, practitioners and teachers, who disparage the practical utility of profound physiological attainments, as an element of professional education.—*Ed. Buffalo Journal.*

"Would it be believed that there are able, nay, scientific, physicians who virtually, at least, prevent the application of physiology to the elucidation of pathology? You occasionally hear half-educated practitioners speak slightingly of him who devotes his leisure to physiological as well as to empirical reading. They hint that he is a "mere reader," "a theorist," "not a practical man," &c., but this is usually in the way of business; it is an ignorant dealer and chapman trying to extinguish a dangerous rival in the true artist. No great harm can result, as the pretender must needs yield at last. Not so, however, with the able physician; such an example I met with not long ago in a weekly journal. The editor, in reviewing a book on practical medicine, had animadverted in a passage, (I think in the introduction) in which the author, an accomplished physician and a physiologist, observed that scientific men are not and cannot be practical, because they have had no experience. The editor was "pulled up" by the offended author, and positively the latter, in his letter of reply to criticism, repeated the offence.

It is obvious enough that a physiologist *and nothing else* cannot be a good physician; but a practitioner may be a physiologist and *something more*. While he studies the experiments of nature, he may have an acute vision for the minute shades of disease, and a quick perception of the effects of medical agents. Nothing will escape his prying eyes. He will be as fertile in curative resources as in theories; as keen a lover of practical information as of scientific knowledge. Further, this practical study of physiology is in itself most interesting. In effecting it you have not the trouble of torturing, and dissecting, and analysing. If, for example, you want illustrations of reflex action, your first patient will probably present them, look closely only. If not seen in your first, they will appear in your second; at all events, wait patiently, and you need not go and catch frogs and snails to vivisect. Indeed you may study the physiology of the nevous system everywhere, in every body; in the man that walks before you in the streets, that stands before you in the pulpit, that struts his hour upon the stage, in people in the ball-room or at a dinner table; nay, you can make your bachelor friend, who pleasantly chats tete-a-tete with you at your fireside, wagging his leg the while, a subject of physiological research. All these, and a thousand more, are good examples of subjects for scientific investigation, presented by persons in perfect health. But when the proteiform diseases of poor human nature come before you, in all their varied phenomena, what an inexhaustible store of matter for observation and meditation.*

* I cannot help giving you a small and homely illustration of these views, which has just occurred to me in looking over Dr. Ranking's excellent Half-yearly Abstract. At p. 72 of vol. ii, you will find two modes of treating scabies, the one the consequence of physiological and microscopical research, the other of empiricism. Now, firstly, scabies gets into very respectable families, and is no jest when a guest with them; 2dly, sulphur, its best cure, stinks. Mr. Stiff, a "*practical* man," and evidently of the right sort, namely, a physiological practitioner, meditates on these two points, and then on the disease. Scabies depends on an insect, the acarus: so the microscope tells us; the acarus belongs to a class of insects that breathe by tracheæ: this entomology sets forth. Comparative physiology shows that (like men), if insects have their tracheæ stopped

You will see that I think a physiological practitioner is the man of the age, and that it is of essential importance to the reformation you advocate that the practitioner should be a thoroughly good physiologist. He should have the science as ready at hand as his snuff-box. He should be *trained* to its use, for every symptom he should at least seek out, if he cannot find, a *rationale*. He should always be curiously catechizing physiology for the why and wherefore. Much might be done in the training of the student by our clinical lecturers, if they would set their shoulders to the wheel; but, unfortunately, the empirical method is the easiest to teach as well as to practice. The student might be led on step by step. He might, for example, first have simple pathological problems in muscular action given to him. A man with morbus coxæ steps across the ward before a clinical class. Problem as follows:—Required to describe the bones and muscles entering into the deformity, and the rationale, physiological and pathological, of its production. Or if the students be more advanced, five patients are brought under the notice of the class; one is in an apopletic fit, a second has scoliosis, a third has valvular disease of the heart, a fourth has phthisis, a fifth, pneumonia. Required to describe the peculiar characteristics of the respiration in each case, and to give a physiological and pathological rationale of them. Would not this method sharpen the diagnostic and therapeutic wits, and be at least one safe and sure step towards a Natural History of Disease? Alas, for the "practical men," those "dealers and chapmen," that sneer in the way of their *meiter* at physiologists as "mere theorists," and "bookworms!" What a rout a few swarms of students so trained would create among them!"

up, they die. Now, Mr. Stiff argued that if the acari were smeared over with an oleaginous substance, their tracheæ would be stopped up, and they would be suffocated; consequently that, after all, it is the lard, and not the stinking sulphur, which cures the itch. He tried the simpler method, and has cured more than forty cases with simple inunction alone. The other example is the *empirical* use of veratria alba, solemnly published without a reason in the Annales de Therapeutique. Now this, as well as half a dozen other *poisons of the acarus*, have been known empirically to vagrants from time immemorial.

